# Is There Still a Place for Surgery in Patients with PCOS? A Review

**DOI:** 10.3390/life13061270

**Published:** 2023-05-28

**Authors:** Luigi Della Corte, Dominga Boccia, Mario Palumbo, Antonio Mercorio, Carlo Ronsini, Giuseppe Bifulco, Pierluigi Giampaolino

**Affiliations:** 1 Department of Neuroscience, Reproductive Sciences and Dentistry, School of Medicine, University of Naples “Federico II”, 80131 Naples, Italy; 2 Department of Public Health, School of Medicine, University of Naples “Federico II”, 80131 Naples, Italy; dominga.boccia@gmail.com (D.B.); mpalumbomed@gmail.com (M.P.); antoniomercorio@gmail.com (A.M.); giuseppe.bifulco@unina.it (G.B.);; 3 Department of Woman, Child and General and Specialized Surgery, School of Medicine, University of Campania “Luigi Vanvitelli”, 80138 Naples, Italy; carlo.ronsini90@gmail.com

**Keywords:** polycystic ovarian syndrome, clomiphene citrate, gonadotrophins, laparoscopic ovarian drilling, transvaginal hydrolaparoscopy

## Abstract

Objective: The surgical management of polycystic ovary syndrome (PCOS) represents an unclear option compared to medical therapy, and it is necessary to deepen the role of minimally invasive surgery, represented by laparoscopic ovarian drilling (LOD) and transvaginal hydrolaparoscopy (THL), for the treatment of PCOS in infertile women resistant to drug therapy and to establish its success in terms of ovulation and pregnancy rates. Methods: A search was performed in the main electronic databases (MEDLINE, EMBASE, Web of Science, PubMed, and the Cochrane Library) from 1994 to October 2022 in order to evaluate the role of surgery in patients with PCOS resistant to pharmacological treatment. Only original scientific articles in English were included. Results: Seventeen studies were analyzed in this review. In all analyzed studies, more than 50% of the population underwent spontaneous ovulation after surgical treatment without great differences between the two surgical techniques (LOD and THL). More than 40% of patients delivered, with a higher rate after LOD, although eight ectopic pregnancies and sixty-three miscarriages were reported. A lower risk of adhesion formation after THL has been reported. No clear data regarding the effect of surgery on the regularization of the menstrual cycle has been described. A reduction in LH and AMH serum levels as well as the LH/FSH ratio compared to preoperative levels for both surgical techniques has been described. Conclusions: Despite the scarcity and heterogeneity of data, surgical therapy could be considered an effective and safe approach in the management of PCOS patients with resistance to pharmacological treatment who desire to become pregnant.

## 1. Introduction

Polycystic ovary syndrome (PCOS), also known as Stein–Leventhal syndrome, is one of the most common diseases among reproductive-age women, with an incidence of 3–15% of all women [1]. The Rotterdam criteria of 2003 are the most commonly used criteria for the diagnosis of PCOS, which is possible if there are two of the three specified conditions: hyperandrogenism, ovulation abnormalities, and/or 12 or more cysts with an ovarian volume > 10 mL [2]. Based on the Rotterdam criteria, four phenotypes of PCOS can be distinguished: HOP with hyperandrogenism (H), ovulation disorders (O), and a polycystic ovary (P) detected by ultrasonography (USG); HO with hyperandrogenism and ovulation disorders but with a normal ovarian at USG; HP with hyperandrogenism and a polycystic ovary at USG but without ovulation disorders; and OP with ovulation disorders and a polycystic ovary at USG but without evidence of hyperandrogenism [3]. Despite these seemingly clear criteria, the etiology of PCOS remains unknown. In this disease, we can find a high ratio of luteinizing hormone (LH) to follicle-stimulating hormone (FSH), which is one of the basic disorders [4]. The underlying causes of PCOS include increased frequency of gonadotropin-releasing hormone (GnRH), increased secretion of LH and anti-Müllerian hormone (AMH), a reduction of FSH concentration, insulin resistance via a post-receptor defect, and obesity that increases the risk of menstrual disorders and hyperandrogenism [5,6,7,8]. This syndrome is considered a multidisciplinary disorder characterized by different symptoms: menstrual disorders (amenorrhea or oligomenorrhea) often leading to ovulation disorders and infertility; acne, alopecia, acanthosis nigricans, and hirsutism; and symptoms of the metabolic syndrome [6,7,8,9,10,11].

The treatment depends on the clinical effect to be obtained: infertility, regulation of menstrual disturbances, reduction of the symptoms of hyperandrogenism, or obesity. For women wishing to conceive, clomiphene is not the first-line therapy but rather letrozole [1,2]. Additionally, metformin and statins, especially for women affected by lipid disorders, as well as gonadotropins, are used to induce ovulation in CC-resistant patients with PCOS [7,8]. Since PCOS patients have a much greater number of follicles in their ovaries, it is important to start with low doses of gonadotropin induction to avoid possible ovarian hyperstimulation syndrome or multiple pregnancies [9,10,11,12,13].

Surgery is a third-line treatment in patients with true resistance to common pharmacological treatments, such as gonadotrophins, or where there is a high risk of multifollicular development and cycle cancellation after gonadotrophin stimulation [1].

The aim of this review is to provide a wide overview of the role of surgery in PCOS patients resistant to pharmacological therapy.

## 2. Materials and Methods

We adhered to the quality standards for narrative reviews, as defined and quantified by “SANRA—a scale for the quality assessment of narrative review articles”. The research was conducted using the following electronic databases: MEDLINE, EMBASE, Web of Science, PubMed, and the Cochrane Library. The studies were identified with the use of a mesh combination of the following keywords: “PCOS”, “polycystic ovarian syndrome”, “clomiphene citrate”, “laparoscopic ovarian drilling”, “LOD”, “transvaginal hydrolaparoscopy”, and ‘’THL’’ from 1994 to October 2022. Two authors (L.D.C. and M.P.) independently screened the titles and abstracts of studies obtained in the search. All types of studies were selected, and each potentially relevant study was obtained in full text and assessed for inclusion independently by the authors. Disagreements were resolved by consensus with a third reviewer (P.G.). Only original papers in English that reported specific experience data on the surgical treatment of PCOS were included. Relevant aspects of every article were recorded and commented on, with particular attention to the modality of treatment applied and the described outcomes. All references were also reviewed by two authors (L.D.C. and D.B.) to avoid missing relevant publications. All reports related to experimental studies conducted on in vitro or animal models were excluded from the analysis. Proceedings of scientific meetings and abstracts were not considered. Two different types of surgery, LOD and THL, used as third-line treatments in PCOS were analyzed [14].

## 3. Results

**Figure 1:**
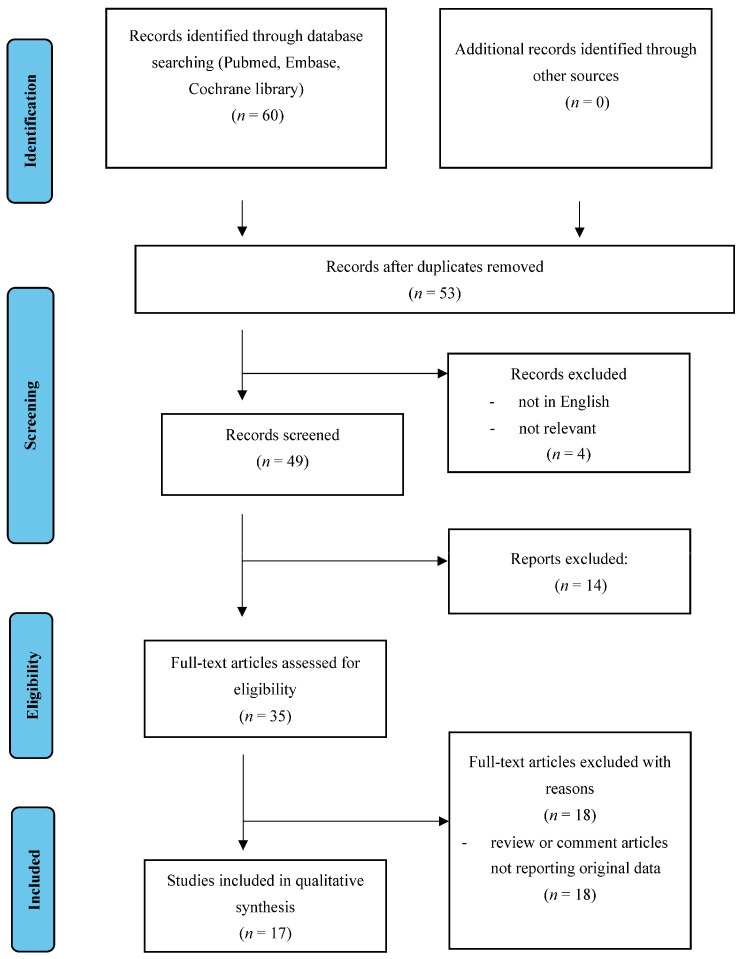
Flow diagram of systematic review search.

Figure 1 illustrates the selection of studies for inclusion in this review. From the bibliographic search, a total of 60 articles were retrieved. Forty-nine articles remained after the first screening. Thirty-two articles were evaluated for eligibility after abstract screening. Finally, 17 studies were included in the study [15,16,17,18,19,20,21,22,23,24,25,26,27,28,29,30,31].

Of the 17 articles included in this review, 12 were used to provide an overview of LOD in CC-resistant patients with PCOS [16,17,19,20,21,22,23,24,25,27,28], with some authors focusing on specific techniques in the laparoscopic field, such as Gjønnaess et al. and Van Wely et al., who reported the use of ovarian electrocautery [15,23], and Duleba et al., the laparoscopic ovarian wedge resection using a harmonic scalpel [20]. Of these, five were retrospective studies [15,16,25,27,28] and eight were prospective ones [17,19,20,21,22,23,24,25]. The further five studies focused on the use of THL [18,25,26,29,30], and of these two were retrospective studies [26,30] and three prospective ones [18,29,31]; one of these compared a group of patients treated with LOD with a group that had undergone THL in terms of ovarian adhesion formation during follow-up [31]. The remaining analysis focused on dosage of hormonal markers and ovarian volume, although data extrapolation was not easy due to the important heterogeneity of the studies published up to now. The younger mean age was 26.4 years old by Kriplani et al. [17], while the “oldest” mean age was 31.25 by Gordts et al. [30]. The characteristics of the included patients are summarized in Table 1.

All women underwent pharmacological treatment and were found to be CC-resistant; therefore, they underwent surgical treatment. However, some of them subsequently underwent, following surgery, other treatments to induce ovulation, such as gonadotropins [17].

To simplify the presentation, the results are divided into two sections based on reproductive outcomes after LOD (Table 2) and after THL (Table 3).

Four studies focused on the menstrual irregularity in PCOS patients and on the possibility of its regulation after laparoscopic surgery (LOD) [15,19,21,22], but there are no clear results. After LOD, a different percentage of women underwent spontaneous ovulation such as 74 [16], 54 [17], 127 [19], 113 [22], 38 [23], and more than 50% [21] patients, respectively; other women ovulated with CC, such as 22 [16], 6 [17], 51 [19], 43 [22], and 21 [23] patients, respectively, or with gonadotropins [17]. In some studies, how ovulation occurred was not specified [15,20,21,24,25,27,28].

Not all women who ovulated became pregnant, and ectopic pregnancy or miscarriage were reported [16,17,19,21,22,28]. This data is not strictly associated with the disease or the treatment related to it, as there is no scientific data to support this relationship. Some studies did not specify if the pregnancy was completed with a delivery or interrupted before [15,20,24,25]. For more details on menstrual cycle pattern, ovulation, and pregnancy rates after LOD, see Table 2.

Regarding the transvaginal surgery, no one analyzed the menstrual cycle pattern. After THL, 12 patients underwent spontaneous ovulation in the study analyzed by Gordts et al., with 25/33 (76%) patients reaching pregnancy, of which 13/25 (52%) with or without controlled ovarian hyperstimulation and/or IVF and 12/25 (48%) directly via IVF [26]. Fernandez et al. reported a lower pregnancy rate (46%) with three patients that spontaneously conceived, two after ovulation induction plus IVF and one after IVF with intracytoplasmic sperm injection [18]; Giampaolino et al. analyzed the ovulation rate one month after treatment (64.1%), after three months (79.5%), and after six months (82.9%) and reported a pregnancy rate of 70.1% [29]; and Gordts et al. reported only the pregnancy rate after THL (25.6%) [30]. Giampaolino et al. reported no surgical complications after both THL and LOD, but after 6 months, 15 (15.5%) patients in the THL group and 73 (70.2%) in the LOD group showed the presence of ovarian adhesion, which indicates that THL ovarian drilling may reduce the risk of ovarian adhesion formation [31]. More details are reported in Table 3.

In addition, a reduction in serum LH levels, LH/FSH ratio, and AMH after surgery was noted, with no great differences regarding the type of surgery used (Table 1 and Table 2).

## 4. Discussion

Due to the complexity of PCOS, the therapy is designed to achieve specific goals such as treating menstrual irregularities, clinical hyperandrogenism, and infertility. Lifestyle changes such as quitting smoking, engaging in physical exercise, and losing weight when necessary are crucial. Oral contraceptive pills (OCP) are typically recommended as the primary treatment for menstrual irregularities since they suppress pituitary LH and reduce testosterone levels, which are usually high in PCOS patients. In addition to OCP, anti-androgens such as cyproterone acetate or spironolactone may also be prescribed to counteract hyperandrogenism and its clinical manifestations [32]. If infertility or subfertility is a concern, it is recommended to undergo an evaluation after 12 months of regular sexual intercourse (2–3 times/week) [33].

The role of PCOS in the development of infertility is linked to the hormonal alteration typical of the pathology in the exam: an increase of LH and a reduction of FSH, closely related to obesity and insulin resistance [34,35]. In addition to stimulating theca cells to produce testosterone, LH acts directly on granulosa cells, reducing the expression of the anti-Müllerian hormone II receptor. This is accompanied by an increase in AMH levels, causing an increase in the number of preantral follicles and small antral follicles, as well as premature induction of an excess of follicular LH receptors, arrest of follicle maturation, and disturbance in the selection of the dominant follicle as a consequence of premature follicular luteinization [36].

The therapeutic procedure in the infertile patient affected by PCOS, to induce ovulation and obtain a pregnancy, foresees the use of clomiphene citrate (CC) as first-line therapy, ovulation induction with gonadotropins as second-line therapy, and surgical treatment as third-line therapy [37,38].

Regarding the differences between the three lines of therapy, the advantages of CC consist of the low risk of pregnancies with more than two fetuses, the low risk of severe OHSS, and the ease of drug administration, while the conventional doses of gonadotropins used in the various protocols for PCOS are associated with a higher risk of ovarian hyperstimulation and multiple pregnancies compared to normal women [39].

In the surgical approaches, there is no risk of ovarian hyperstimulation, the incidence of multiple pregnancies is the same as in spontaneous conceptions in ovulatory women, and there is no need for monitoring, unlike in the CC, which must be monitored (by ultrasound and endocrine blood sample) to appreciate the day of the ovulation and to measure follicular growth and endometrial thickness [40].

Surgery may be the next step in treatment for patients with PCOS in cases of resistance to pharmacological therapy. To describe resistance to the induction of ovarian stimulation with drug therapy, a clarification of the pathogenesis of PCOS is important. Androgen biosynthesis is mediated by microsomal P450c17, which catalyzes the activity of 17–20 lyases. P450c17 transcriptional and post-transcriptional alterations have been implicated in the etiology of PCOS. Indeed, these women show a relative inhibition of 17–20 lyase activity relative to 17-hydroxylase, leading to an increase in the 17OHP/A ratio. Administration of GnRH or hCG in women with PCOS causes excessive production of 17OHP. Low aromatase activity has also been demonstrated in women with PCOS. It may be partly responsible for the hyperandrogenism in this syndrome. Elevated androgen levels can negatively impact follicular development, causing atresia, and ovarian development, inhibiting meiotic maturation by decreasing intracytoplasmic calcium level fluctuations. We therefore clarify how testosterone induces insulin resistance in female adipocytes and impairs insulin-mediated glucose uptake through the activation of selective metabolic signaling pathways and androgen receptor alterations [41].

The most commonly used approaches are LOD, with the creation of holes on the ovarian surface through different sources of energy such as laser, monopolar current, or bipolar current, and THL, which offers a valuable alternative to the standard laparoscopic procedure through a needle puncture of the posterior vaginal fornix with a miniaturized bipolar needle. The choice of surgery in the treatment of PCOS is debated mostly for the drawback of adhesion formation. Therefore, some authors prefer to limit it to patients who do not respond to CC in a dose of 200 mg/day for 5 days or to long-term gonadotropin analogues, those that are hyperresponsive to gonadotropin therapy as second-line therapy, or those that require a diagnosis for infertility. The advantages of surgery include a durable effect, monofollicular cycle restoration, and a reduced incidence of miscarriage [24,25,26]. The results obtained with THL are comparable with those obtained with standard laparoscopy, as reported by Fernandez et al., which are described as equivalent, with a lower risk of developing adhesions for THL [18]. A potential complication of transvaginal access is rectal perforation. Verhoeven et al. reported a 0.5% incidence, and in a survey of 3667 procedures, all cases except one were managed conservatively with antibiotics. The transvaginal approach is recommended to exclude disease of the pouch of Douglas, and the presence of bleeding, infection, and a large ovarian cyst are contraindications for this approach [3,42,43,44]. After surgery, it is possible to undergo hormonal stimulation or assisted reproduction treatments, such as in nine patients after LOD [21] and twelve after THL [30] who underwent in vitro fertilization (IVF). Women are often referred to IVF because of aberrant semen parameters or a history of previously failed procedures, as reported by Gordts et al. [30]. Assisted reproductive technology (ART) therapies, such as IVF and intracytoplasmic sperm injection (ICSI), are a valid option only in the presence of failure of therapies for inducing ovulation because the risk of excessive response to FSH stimulation and hence subsequent development of ovarian hyperstimulation syndrome is quite high [32].

The two surgical techniques have been compared in several aspects. Giampaolino et al. compared the length of the procedure in two groups of patients (LOD vs. THL group), and it was significantly shorter in the THL group (20 ± 10 vs. 40 ± 10 min, respectively) [31]. Concerning complications, the same study reported no intra- and immediate post-operative complications in both groups (LOD vs. THL). Six months after these procedures, on 201 patients (45 patients were lost to follow-up), the evaluation of ovarian adhesion was performed. The analysis showed that 15 (15.5%) patients in the THL group and 73 (70.2%) in the LOD group showed the presence of any type of ovarian adhesion. In particular, eight (53.3%) and forty-four (60.3%) patients showed filmy adhesions, five (33.3%) and twenty-two (30.1%) dense adhesions, and two (13.3%) and seven (9.6%) cohesive adhesions in the THL and LOD groups, respectively. No multiple pregnancies were observed, and no differences in cumulative pregnancies were detected between the two groups. The pregnancy rate was evaluated as a cumulative rate of 68% for both groups [31].

The role of surgery in the regulation of the menstrual cycle is still unclear. Some authors have reported a great regularization of the menstrual cycle after surgery [16,19], others not [22,28], but the data remain very heterogeneous and often poorly defined.

Regarding ovulation, there was no significant difference in ovulation induction between LOD and THL; in more than 50% of the patients included in the analysis, it was spontaneously obtained. However, in a small sample of patients, it was obtained through CC or gonadotropins after surgery, as reported by Kriplani et al., where in six patients (9.1%), ovulation was obtained with CC and in two (3%) with gonadotropins out of fifty-four patients (81.8%) spontaneously [17].

In all analyzed studies, pregnancy was obtained after surgery, without a significant difference between LOD and THL [15,16,17,18,19,20,21,22,23,24,25,26,27,28,29,30,31]. In some cases, issues arose after the implantation of the blastocyst, and not all obtained pregnancies ended with delivery; on the whole, eight were ectopic pregnancies, three were spontaneous abortions, and sixty were miscarriages [16,17,19,21,22,28]. At present, due to the poor practical application of surgery in PCOS and the difficulty in establishing adequate follow-up, there is insufficient evidence to understand the reason.

Another difference concerns the postoperative pain complained of by the patient after both procedures (LOD and THL) to be evaluated with a postoperative pain VAS score: it turned out to be significantly higher in women who underwent LOD compared to THL (3.26 ± 1.1 for LOD vs. 1.11 ± 0.5 for THL) [13].

Both LOD and THL ovarian drilling did not induce any change in serum FSH levels during the 6 month follow-up period, while serum LH levels and the LH/FSH ratio were significantly reduced in comparison with baseline in both approaches, but not significantly, likely correlated with an increased loss of the cauterized ovarian stroma during both procedures. In addition, we know that PCOS is characterized by high serum AMH levels compared with healthy women, correlated to the increased number of preantral and small antral follicles. While preoperative serum AMH levels were similar in both groups, postoperative levels were significantly reduced compared to preoperative ones (6.06 ± 1.18 and 5.84 ± 1.16 vs. 5.00 ± 1.29 and 4.83 ± 1.10 for LOD and THL, respectively). Both techniques, therefore, determined a marked decline in these abnormally elevated AMH concentrations. This finding may indicate that the mechanisms of action of THL ovarian drilling are similar to those occurring with LOD. It is not clear if it is a temporary normalization of ovarian markers with subsequent recovery or a permanent reduction in ovarian reserve [13]. At present, due to the heterogeneity of the studies, there is insufficient evidence to prove that.

## 5. Conclusions

Surgical therapy, combined or not with pharmacological ones, could be considered an effective and safe approach in the management of PCOS women who desire to have a pregnancy. Furthermore, the different surgical options that characterize the management of PCOS should be compared to identify an optimal treatment and improve reproductive outcomes with ovulation and pregnancy rates.

There was no significant difference in ovulation induction between LOD and THL; in more than 50% of the patients included in the analysis, ovulation was spontaneously obtained. No multiple pregnancies were observed, and no differences in cumulative pregnancies were detected between the two groups (LOD and THL). Different authors described a great regularization of the menstrual cycle after surgery, both LOD and THL, but the data remained very heterogeneous. Both techniques did not induce any change in serum FSH levels during the 6 month follow-up period, while serum LH levels and the LH/FSH ratio were significantly reduced, as were the AMH concentrations. The included studies reported no intra- and immediately postoperative complications in both groups, but six months after these procedures, ovarian adhesion was more prominent in the LOD group.

However, long-term prognosis data, including the recurrence of disease, the trend of ovarian reserve, the development of long-term complications, and the progress of the potential pregnancy to term, are needed, as well as a longer follow-up period because some patients have been lost.

The mechanisms by which a patient does not respond to ovarian stimulation are still to be clarified, especially in such complex conditions as PCOS.

In conclusion, the surgical treatments of PCOS in patients with resistance to pharmacological treatment need further larger series and randomized clinical trials to assess the effectiveness and safety of such combined treatments because, to date, the studies on which our results are based have largely been retrospective, which is a weakness for our results.

## Figures and Tables

**Table 1:** Characteristics of the included studies and patients (NS: not specified; NR: not reported; CC: clomiphene citrate; LOD: laparoscopic ovarian drilling; THL: transvaginal hydrolaparoscopy; LH: luteinizing hormone; and FSH: follicle stimulating hormone).

Study	Country	Type of Study	Sample Size, n°	Age (Years—Mean ± SD or Median (Range))	Symptoms and Signs (n—%)	BMI (kg/m^2^) (Mean ± SD)	Medical Treatment, (n)	Surgical Treatment	Hormonal Levels in Plasma before Treatment (Mean ± SD or Median (Range))					Ovarian Volume (mL—Mean ± SD)
									LH (IU/L)	FSH (IU/L)	LH:FSH Ratio	AMH (ng/mL)	Testosterone (nmol/L)	
Gjønnaess, 1994 [15]	Norway	Retrospective study	109	NR	NS: oligomenorrhea and cystic glandular hyperplasia of the endometrium	58.7 ± 6.2	CC (109)	Ovarian electrocautery	14.6	6.3	NR	NR	2.7	NR
Li et al., 1998 [16]	Japan	Retrospective study	111	28.6 ± 3.9	24 (22) hirsutism 80 (72) oligomenorrhea 17 (15) amenorrhea	26.0 ± 4.8	CC (87)	LOD	15.4 ± 9.1	5.4 ± 1.9	2.5 ± 2.1	NR	NR	NR
Kriplani et al., 2001 [17]	India	Prospective study	70	26.4	59 (84.3) oligomenorrhea 11 (15.7) secondary amenorrhea	<29 kg/m^2^ in 42 (60%)	CC (70)	LOD	>10 IU/L in 42 (60%)	NR	>2 in 32 (45.7%)	NR	NR	NR
Fernandez et al., 2001 [18]	France	Prospective study	13	28.5 ± 3.9	2 (15.3) hirsutism 3 (23.07) oligomenorrhea 1 (7.69) amenorrhea	24.1 ± 4.5	CC (12)	THL	NR	NR	2.0 ± 0.8	NR	NR	NR
Al Ojaimi, 2003 [19]	Asia	Prospective study	198	30.5 ± 5.7	132 (66.7) oligomenorrhea 41 (20.7) amenorrhea	29.8	CC (184) and additional gonadotrophin therapy (72)	LOD	13.3 ± 5.6	6.1 ± 3.0	2.3 ± 1.1	NR	2.4 ± 1.3	NR
Duleba et al., 2003 [20]	Poland	Prospective study	33	26.8	NS: oligomenorrhea, hirsutism, or acne	29.3	NR	Laparoscopic ovarian wedge resection using harmonic scalpel	13.4	5.9	2.33	NR	0.8	12.6
Stegmann et al., 2003 [21]	Arizona	Prospective study	86	30.2	49 endometriosis 54 adhesions NS hirsutism NS acanthosis	29.35	CC (NS)	LOD	NR	NR	1.94	NR	17.39	4.2 (median left ovary) 4.3 (median right ovary)
Amer et al., 2004 [22]	UK	Prospective study	200	28.9 [3.9]	149 (74) oligomenorrhoea 36 (18) amenorrhea 70 (35) hirsutism	27.1 ± 4.9	CC (200)	LOD	14.2 ± 6.6	5.2 ± 1.4	2.8 ± 1.2	NR	2.6 ± 1.2	11.4 ± 3.6
Van Wely et al., 2005 [23]	Netherlands	Prospective study	83	28.5 [1.0]	35 (42.1) amenorrhea 47 (56.6) oligomenorrhea	27 ± 6.2	CC (83)	Ovarian electrocautery	NR	NR	NR	NR	NR	NR
Palomba et al., 2006 [24]	Italy	Prospective study	60	29.3 [5.2]	NS	27.6 ± 1.9	CC (60)	LOD	18.0 ± 4.1	8.9 ± 3.4	NR	NR	3.8 ± 1.2	NR
Amer et al., 2009 [25]	UK	Prospective study	47	28.4 [0.9]	NS—oligo/amenorrhea and/or hyperandrogenemia	26.9 ± 0.6	CC (18)	LOD (29/47)	11.7 (4.6–23.7)	5.1 (2.9–8.2)		6.1 (1.0–21.0)	2.5 (1.0–4.0)	11.9 ± 1.1
Gordts et al., 2009 [26]	Belgium	Retrospective study	39	30.4 [3.8]	NS—amenorrhea or oligomenorrhea, with or without hirsuitism	29.4 ± 9.7	CC (16)	THL	15 ± 10	5.5 ± 2	2.7	NR	1.7 ± 1.2	NR
Ott et al., 2009 [27]	Austria	Retrospective cohort study	100	28.2	NS—anovulation, oligomenorrhea, hirsutism, acne, and infertility	26.5	CC (100)	LOD	15.1 (6.0)	6.2 (1.7)	NR	NR	0.8 (0.4)	NR
Kaur et al., 2013 [28]	India	Observational retrospective study	100	27 [3.2]	NS- anovulation, oligomenorrhea, hirsutism, acne, and infertility	26.6 ± 4.2	CC (100)	LOD	NR	NR	2.1 (1.1)	NR	NR	NR
Giampaolino et al., 2016 [31]	Italy	Prospective randomized study	123	27.5 ± 6.8	NR	27.3 ± 5.6	CC (123)	THL	NR	NR	NR	5.84 ± 1.16	1.2 ± 0.3	NR
			123	30.1 ± 7.5	NR	25.9 ± 7.1	CC (123)	LOD	NR	NR	NR	6.06 ± 1.18	1.6 ± 0.2	NR
Giampaolino et al., 2017 [29]	Italy	Prospective observational study	117	29.5 [3.9]	NS—oligomenorrhea and amenorrhea, hyperandrogenism, acne, hyrsutism, and androgenic alopecia	NR	CC (117)	THL	8.85 (1.39)	5.35 (0.71)	1.68 (0.35)	NR	NR	11.78 (1.61)
Gordts et al., 2021 [30]	Belgium	Retrospective cohort study	2288	31.25 [3.8]	366 (15.9) endometriosis 144 (6.3) adhesions	-	CC (NS)	THL	NR	NR	NR	NR	NR	NR

**Table 2:** Outcomes of laparoscopic ovarian drilling (LOD) [CC: clomiphene citrate; IVF: in vitro fertilization; NR: not reported; * comparison between conception and non-conception groups; ** three groups of patients with the value of hormonal level and number of patients for group (n); *** four groups of patients divided by age: A (<25 y), B (25–30 y), C (30–35 y), and D (>35 y)].

	Menstrual Cycle Pattern, n (%)	Ovulation, n (%)	Pregnancy Outcomes, n (%)	Hormonal Levels in Plasma Post-LOD Treatment Mean ± SD or Median (Range)				
				LH (IU/L)	FSH (IU/L)	LH/FSH Ratio	AMH (ng/mL)	Testosterone (nmol/L)
Gjønnaess 1994 [15]	NR	105 (96.3)	76 (69.7)	NR				
Li et al., 1998 [16]	160 (88) regular 23 (21) irregular	74 (67) spontaneously 22 (20) via CC 15 (13) none	58 (52) ongoing/delivery 7 (6) miscarriages 2 (2) ectopic 44 (40) none	7.7 (1) vs. 10 (1.3) *	5.6 (0.6) vs. 6.9 (1.7) *	NR	NR	NR
Kriplani et al., 2001 [17]	NR	54 (81.8) spontaneously 6 (9.1) via CC 2 (3) via gonadotropins	50 (71.4): − 42 (84) delivery − 8 (16) miscarriages	NR				
Al Ojaimi et al., 2003 [19]	160 (88.4) regular 21 (11.6) irregular	127 (70.1) spontaneously 51 (28.2) via CC 3 (1.7) none	120 (66.3) ongoing/delivery 31 (17.2) miscarriages 2 (1.1) ectopic 28 (15.5) none	13.3 (5.6)	6.1 (3.0)	2.3 (1.1)	NR	NR
Duleba et al., 2003 [20]	NR	NR	22 (67)	8.9 (1.9)	5.8 (0.6)	1.4 (0.3)	NR	0.47 (0.04)
Stegmann et al., 2003 [21]	57 (66) regular	(>50) spontaneously	43 (89.6), of which 9 by IVF 1 (2.1) ectopic 1 (2.1) triplet gestation abortion 3 (6.2) miscarriages	NR				
Amer et al., 2004 [22]	15 (8) regular 185 (92) irregular	113 (57) spontaneously 43 (21) by CC 44 (22) none	86 (45) ongoing/delivery 9 (4) miscarriages 2 (1) ectopic 96 (50) none	<10 (55) 10–19.9 (99) ≥20 (39) **	NR	>2 (51) 2–3.9 (108) ≥4 (32) **	NR	6 (58) 6–4.49 (49) ≥4.5 (9) **
Van Wely et al., 2005 [23]	NR	38 (46) spontaneously 21 (25) via CC 24 (29) none	41 (49) ongoing/delivery	11.5 (5.6)	6.1 (2.1)	2.0 (9.6)	NR	4.0 (1.7)
Palomba et al., 2006 [24]	NR	3 (0.5%)	33 (60)	A 19.0 (3.7) B 17.6 (4.2) C 18.1 (3.9) D 18.4 (4.9) ***	A 7.6 (1.6) B 8.6 (3.1) C 9.3 (3.4) D 10.6 (4.7) ***	NR	NR	A 3.7 (1.2) B 3.9 (3.2) C 3.8 (1.1) D 3.8 (1.3) ***
Amer et al., 2009 [25]	NR	24 (83) 5 (17) none	15 (52)	7.9 (1.9–21.0)	5.5 (1.7–7.7)	NR	4.3 (0.3–15.1)	2.2 (1.1–3.6)
Ott et al., 2009 [27]	NR	71 (71) 29 (29) none	36 (36) ongoing/delivery	6.4 ± 4.0	NR	NR	NR	NR
Kaur et al., 2013 [28]	18 (18) regular 82 (82) irregular	NR	35 (85.4) ongoing/delivery 5 (12.2) miscarriages 1 (2.4) ectopic	NR	NR	2.1 ± 1.1	NR	NR
Giampaolino et al., 2016 [31]	NR	NR	(68—cumulative rate)	NR				

**Table 3:** Outcomes of transvaginal hydrolaparoscopy (THL) [IVF: in vitro fertilization; LPS: laparoscopy; and CC: clomiphene citrate].

	Ovulation, n (%)	Pregnancy, n (%)	Hormonal Levels in Plasma Post-THL Treatment Mean ± SD or Median (Range)				
			LH (IU/L)	FSH (IU/L)	LH/FSH Ratio	AMH (ng/mL)	Testosterone (nmol/L)
Fernandez et al., 2001 [18]	6/13 (46) regular 5/13 (39) amenorrhea 2/13 (15) immediately pregnant	6/13 (46): − 3 spontaneously − 2 after ovulation induction plus IVF − 1 after IVF with intracytoplasmic sperm injection	NR				
Gordts et al., 2009 [26]	12/28 (43) spontaneously 16/18 (57) by CC plus hMG	25/33 (76) − 13/25 (52) natural conception with or without controlled ovarian hyperstimulation and/or IVF − 12/25 (48) after IVF	8 ± 3	6.1 ± 1.5	1.3	NR	1.1 ± 0.7
Giampaolino et al., 2016 [31]	NR	(68—cumulative rate)	NR				
Giampaolino et al., 2017 [29]	64.1% after 1 month 79.5% after 3 months 82.9% after 6 months	(70.1)	NR				
Gordts et al., 2021 [30]	NR	(25.6)	NR				

## Data Availability

The present review was based on published articles. All summary data generated during this study are included in this published article. Raw data used for the analyses are available and presented in the original reviewed articles.
